# Environmental enrichment reverses stress-induced changes in the brain-gut axis to ameliorate chronic visceral and somatic hypersensitivity

**DOI:** 10.1016/j.ynstr.2023.100590

**Published:** 2023-11-24

**Authors:** A. Orock, A.C. Johnson, E. Mohammadi, B. Greenwood-Van Meerveld

**Affiliations:** a Department of Physiology, University of Oklahoma Health Sciences Center, Oklahoma City, OK, USA; b Department of Veterans Affairs Health Care System, Oklahoma City, OK, USA

**Keywords:** Stress, Visceral sensitivity, Brain-gut communication, Colon permeability somatic sensitivity, Behavioral therapy

## Abstract

**Introduction:**

Behavioral therapies, including cognitive behavioral therapy, hypnotherapy and stress management activities, have emerged as effective treatments for irritable bowel syndrome (IBS), a female predominant disorder of the brain-gut axis. IBS, affecting over 10% of the global population, typically presents with abnormal bowel habits and abdominal pain due to visceral hypersensitivity. While the mechanisms underlying how behavioral therapies treat IBS are still elusive, we had previously shown that chronic stress alters gene expression in brain regions critical for stress processing and nociception. We found that exposure to an enriched environment (EE), the rodent analogue of behavioral therapies, prior to and during the stressor was sufficient to prevent stress-induced changes in glucocorticoid receptor (GR) expression in the central nucleus of the amygdala (CeA) and hippocampus. Pre-exposure to EE also inhibited stress-induced increased colonic permeability and was able to block the induction of stress-induced visceral and somatic hypersensitivity. However, it remains unknown if EE can reverse chronic viscerosomatic hypersensitivity that persists following exposure to stress. We hypothesized that EE after chronic stress would be sufficient to reverse stress-induced changes in i) GR expression in the CeA and hippocampus, ii) ameliorate stress-induced colonic hyperpermeability and iii) restore normal visceral and somatic sensitivity in male and female rats.

**Methods:**

Male and female rats were exposed to daily water avoidance stress (WAS). After confirming the rats had developed visceral hypersensitivity, 50% of the animals were housed in EE for 2 weeks while the other 50% remained in standard housing (SH). At the end of this period, we assessed visceral and somatic sensitivity. We also collected colon tissue to measure colonic permeability. Micro-punches of tissue from the CeA and hippocampus were isolated to measure GR expression. Control animals not exposed to WAS were kept in SH for the duration of the study (n = 8 per group).

**Results:**

In both male and female rats, EE reversed stress-induced visceral (p < 0.001) and somatic (p < 0.01) hypersensitivity when compared to WAS animals housed in SH to levels comparable to control animals. EE exposure also reversed changes in GR expression in both the hippocampus (p < 0.01) and CeA (p < 0.01), normalizing GR expression to control levels. EE exposure ameliorated stress-induced colonic hyperpermeability in both male (p < 0.01) and female (p < 0.01) rats compared to WAS rats in SH.

**Conclusion:**

Our findings suggest that behavioral therapies are viable therapeutic options for IBS as they can counter the stress-induced pathophysiology underlying IBS symptoms including visceral hypersensitivity, increased colonic permeability and altered gene expression.

## Highlights

• Stress is a major trigger for the symptoms of irritable bowel syndrome (IBS).
• Stress disrupts glucocorticoid receptor (GR) expression in hippocampus and amygdala.
• Environmental enrichment (EE) can reverse stress-induced colonic hyperpermeability.
• EE reverses stress induced changes in GR expression to control levels.
• Exposure to EE reversed visceral and somatic hypersensitivity in males and females.

## 1 Introduction

Behavioral therapies are a group of interventions that have historically been used to treat mental health disorders including psychiatric, anxiety and other mood disorders (David et al., 2018; Zimmermann et al., 2005; Bisson et al., 2007). These interventions typically aim to identify maladaptive and harmful behaviors and patterns that contribute to the development of symptoms and then use tailored cognitive and behavioral techniques to counteract these and in the process, ameliorate symptoms (Orock et al., 2021a). Behavioral therapies including cognitive behavioral therapy (CBT), hypnotherapy, and various stress relief techniques have also been shown in clinical studies to alleviate various types of pain including inflammatory, neuropathic (Otis et al., 2013) and musculoskeletal (Castro et al., 2012). Behavioral therapies including CBT have also shown efficacy in treating visceral pain in patients with irritable bowel syndrome (IBS) (Lackner et al., 2018; Tang et al., 2013) and inflammatory bowel disease (IBD) (Chen et al., 2021; Gracie et al., 2017).

IBS is a disorder of the brain-gut axis characterized by abnormal bowel habits and visceral hypersensitivity that manifests as chronic visceral pain (Chang et al., 2009). IBS affects about 10–20% of the US population (Peery et al., 2012) and displays a female-predominant phenotype as there are twice as many female as male patients (Kim et al., 2018). IBS symptoms have many potential causes; however, stress has emerged as a key trigger for IBS symptoms (Jankord et al., 2008; Meerveld et al., 2018). The most common comorbidities of IBS are other stress and anxiety related disorders (Banerjee et al., 2017) as well as pain disorders such as fibromyalgia (Yang et al., 2015) and bladder pain syndrome (Doiron et al., 2017). Most pharmacological therapies developed for IBS focus on alleviating symptoms or restoring normal bowel function without accounting for the multifactorial nature of the disorder. As a result, these drugs are not effective in all patients and may only serve as temporary relief from symptoms highlighting the need for more comprehensive treatment approaches (Orock et al., 2021a; Quigley et al., 2018). Due to the effectiveness of behavioral therapies in many other stress-related disorders and the role of stress in worsening IBS symptoms, clinicians began investigating whether behavioral therapies could alleviate the symptoms of IBS and whether countering the effects of stress directly could lead to longer lasting symptom relief. While many clinical studies have shown that behavioral therapies can ameliorate IBS symptoms (Lackner et al., 2018), the underlying mechanisms of action are not understood.

Imaging studies have shown that the amygdala, a region of the brain that regulates the body's neuroendocrine response, is hyperactive during colorectal distensions in IBS patients when compared to healthy controls (Wilder-Smith et al., 2004). During stressful periods, cortisol, the main stress hormone, activates the amygdala to regulate the hypothalamic-pituitary-adrenal (HPA) axis and its downstream effects (Chang et al., 2009). However, a hyperactive stress response can alter how the brain processes various sensory signals including nociception and can exacerbate IBS symptoms including chronic pain. Other brain regions, including the hippocampus, are known to negatively regulate the HPA axis under normal stress conditions, however, under chronic stress, persistent glucocorticoid receptor (GR) activation also reduces the negative feedback effect of the hippocampus on the stress axis (Jankord et al., 2008). Our preclinical studies have shown that the central nucleus of the amygdala (CeA) plays a major role in the pathophysiology of stress-induced visceral pain. Stereotaxic placement of corticosterone micropellets on the CeA of rats was sufficient to induce visceral hypersensitivity (Greenwood-Van Meerveld et al., 2001) and decrease the expression of GR. We also showed that repeated water avoidance stress (WAS) was sufficient to decrease GR expression in the CeA and increase colonic permeability to cause visceral hypersensitivity in rats (Orock et al., 2021b). Subsequent studies showed that decreasing GR expression in the CeA using antisense oligodeoxynucleotides was sufficient to induce visceral hypersensitivity (Johnson et al., 2015), highlighting the importance of GR expression in the development of stress-induced visceral hypersensitivity. Stress also affects the gut by causing increased permeability, which allows the translocation of luminal contents causing sensitization of afferent neurons leading to increased pain perception (Orock et al., 2021b). Visceral nociception can also sensitize somatic afferent fibers as these signals converge at the level of the dorsal root ganglia (Mohammadi et al., 2018). This cross sensitization of visceral and somatic afferents could explain why IBS patients may also present with comorbid somatic pain disorders (Ustinova et al., 2006). In support of this concept, we showed that stress also induced somatic hypersensitivity in the WAS model (Orock et al., 2020).

Environmental enrichment (EE) is a rodent analogue of behavioral therapies widely used in preclinical studies to reveal the molecular mechanisms of behavioral therapies (Skwara et al., 2012; Girbovan et al., 2013). EE is defined as housing animals in an environment with sufficient physical and social stimuli to enhance brain stimulation and activity (Sampedro-Piquero et al., 2017). Our previous studies in both male and female rats showed that pre-exposure to an EE before and during repeated WAS was sufficient to inhibit stress-induced reduction in GR expression in the CeA. EE also blocked the stress-induced increase in colonic permeability and prevented the development of stress-induced visceral and somatic hypersensitivity (Orock et al., 2020, 2021b). While our previous data revealed the potential underlying mechanisms via which exposure to EE *before* the stressor (pre-dosed) prevents the development of stress-induced visceral hypersensitivity, it is not clear whether EE can reverse stress-induced visceral hypersensitivity in a more clinically relevant situation when the disorder is already established by once again stabilizing GR expression in the CeA and restoring colonic integrity. Clinical and preclinical studies indicate that increased colonic permeability also plays a role in the development and maintenance of visceral hypersensitivity in IBS (Mohammadi et al., 2018; Camilleri et al., 2012) but it is unclear whether behavioral therapies can also influence colonic permeability. In this study, we hypothesized that housing animals previously exposed to repeated water avoidance stress could reverse stress-induced changes in GR expression and colonic permeability to ultimately ameliorate stress-induced visceral and somatic hypersensitivity.

## 2 Methods and experimental design

### 2.1 Ethical statement and assurances

All procedures used were approved by the Veterans Affairs (VA) Health Care System (1809-001, 2110-001) and the University of Oklahoma Health Sciences Center (OUHSC) (21-023-SAFHI) institutional animal care and use committees (IACUCs). Experiments were designed to minimize pain and distress to the animals per the Guide for Care and Use of Laboratory Animals (8th edition, 2011).

### 2.2 Animals

This study was performed with adult male (250–300 g) and female (150–180 g) 74–90 days old Fischer-344 (F344) rats (Charles River Laboratory, Wilmington MA, USA). The animals were maintained on a 12 h light/dark cycle (lights on at 07:00) at 21 °C with ad libitum access to food and water. Animals were acclimated to the animal facility and behavioral laboratory for 1 week each before the start of the experiments.

## 3 Experimental procedure

After acclimatization in the behavioral lab, rats (both males and females) were randomly (www.randomization.com) divided into 3 groups (n = 8/group/sex); sham controls, stressed animals to be housed in standard housing (SH + WAS) and stressed animals to be housed in an enriched environment (EE + WAS). Animal numbers were chosen based on power analysis and previous published studies (Orock et al., 2021b). The stress groups were exposed to once daily water avoidance stress (WAS) for 7 days. Control animals were placed in SHAM chambers. At the end of the WAS/SHAM procedure (on day 8), we assessed visceral and somatic sensitivity in these animals to show that the WAS animals were hypersensitive. One week after the visceral sensitivity assessment, half of the hypersensitive animals were placed in enriched housing (EE + WAS) for 2 more weeks. The rest of the WAS and control animals stayed in standard housing (SH). After 2 weeks of EE (on day 28), visceral and somatic sensitivity were once again measured. In a separate cohort of animals that did not undergo colorectal distension (which can compromise colon integrity), we collected colon tissue to measure permeability. We also collected CeA and hippocampal tissue to measure protein levels.

### 3.1 Water avoidance stress

We performed the WAS procedure as previously described (Orock et al., 2021b). Briefly, male and female rats were placed on a small platform (8 × 8 × 8 cm) mounted in the center of a semi-transparent plastic container which had been filled with room temperature water to about 1 cm below the surface of platform. Control animals are placed in SHAM containers which are identical to those used for the WAS animals, but these did not have any water and the animals were free to move about the container. The animals were weighed daily before being left undisturbed in the containers for 1 h. The animals were then returned to their home cages and the fecal pellet output (FPO) during that hour was counted as a measure of stress-induced autonomic output.

### 3.2 Environmental enrichment and standard housing procedures

Standard housing (SH) and environmental enrichment (EE) were used as previously described (Orock et al.). To provide positive physical enrichment, large cages (78 cm L x 52 cm W x 100 cm H) were used to house EE rats. These cages had sufficient bedding, toys (different shapes and sizes of wood chews and sticks; bio-serv.com), enriched diet (sweetened cereal, seed/nut mix), and burrowing tunnels to keep the rats entertained. We also placed extra cage-mates in the EE cages (4 animals per cage) to account for social enrichment. SH control animals were housed (2 per cage) in shoe-box cages with filter tops (43 cm L x 20 cm W x 21.5 cm H). Both housing conditions were kept in the same room in the animal facility.

### 3.3 Visceral sensitivity assessment

We measured the visceromotor response (VMR) to colorectal distensions (CRD) in freely moving rats as a measure of visceral sensitivity using the protocol previously described (Orock et al.; Tran et al., 2014). Briefly, rats were fasted overnight and while under anesthesia (2–2.5% isoflurane), we inserted a balloon catheter (made with the tip of a latex condom) approximately 10 cm into the distal colon via the anal cavity and fixed the catheter in place with surgical tape around the tail and the animals were allowed 30 min to recover from the anesthesia (Plourde et al., 1997). Rats were not shaved to avoid any confounding effects to their behavior due to the change in their coat during the assay. We induced CRD by inflating the balloon to isobaric pressures of 20, 40 and 60 mmHg for 10 min each (the order of the distension pressures was randomized between animals), with 10 min of rest (0 mmHg) between distensions using a barostat (G&J Electronics, Toronto, ON, Canada) (Al-Chaer et al., 2000). VMR was measured visually as the number of abdominal contractions resulting in the animal arching its back, lifting its abdomen, vocalizing, freezing, jumping, or any combination of those behaviors during these distensions. The experimenter was blinded to the SHAM and WAS animal groups.

### 3.4 Somatic sensitivity assessment

Somatic sensitivity, calculated as the minimal force required to elicit a withdrawal reflex on the hind paw of the rats, was measured as previously described (Orock et al., 2021b; Orock et al.). Briefly, rats were placed in an elevated Plexiglas box with a wire mesh floor and allowed to acclimate for 10 min. The IITC 2390 series Electronic von Frey Anesthesiometer (IITC Life Science, Woodland Hills, CA) was then used to apply increasing force to the plantar region of the hind paw until a withdrawal reflex was observed. The minimum force required to elicit this reflex was recorded and the process was done 4 times total with 5-min intervals between each session. The mean value of the 4 trials was used for the withdrawal threshold. The experimenter was blinded to the SHAM and WAS animal groups.

### 3.5 Tissue processing and Western blot analysis

We took a separate cohort that underwent WAS/SHAM and EE/SH but did not undergo CRD and on day 28 post beginning of the WAS, these animals were deeply anesthetized and euthanized. We collected brain punches of the central nucleus of the amygdala (CeA) and a piece of the hippocampus dorsal to the CeA from the animals for Western blot processing. The brain tissue was lysed in Pierce™ RIPA buffer (ThermoFisher) containing Halt™ protease (ThermoFisher.com) and Halt™ phosphatase (ThermoFisher.com) inhibitor cocktails and protein concentrations were obtained using a Pierce™ BCA Protein Assay Kit (ThermoFisher.com). Equal amounts of protein were loaded into the automated “Sally Sue” system (bio-techne) to measure changes in protein expression according to the protocol in the “12–230 kDa Separation Module for Sally Sue Systems” kit (www.bio-techne.com). All antibodies used were from commercial sources (cell signaling technology) with well-established and public validation data as indicated by the catalogue numbers. We used glucocorticoid receptor (D8H2) XP (1:50, Rabbit mAb #3660) for the primary antibody, GAPDH (14C10) (1:50, Rabbit mAb #2118) as the housekeeping antibody and anti-rabbit IgG, (1:100, HRP-linked Antibody #7074) as the secondary antibody (www.cellsignal.com). The sally sue system uses capillary electrophoresis instead of gels to identify and quantify proteins of interest and these are then digitally represented as bands on a virtual gel. The experimenter was blinded to the animal groups.

### 3.6 Colonic permeability assessment

Each rat's colon was collected postmortem on day 28 from the cohort which did not undergo CRD and placed into oxygenated (with 95% O_2_–5% CO_2_) ice-cold Krebs buffer composed of 120 mM NaCl, 6 mM KCl, 1.2 mM MgCl_2_, 1.2 mM H_2_PO_4_, 2.5 mM CaCl_2_, 14.4 mM NaHCO_3_, and 11.5 mM glucose. The tissue was opened longitudinally and the muscle layer and attached myenteric plexus were gently peeled off and mucosal sheets were clamped between two modified Ussing chambers. Tissues were bathed in oxygenated Kreb's solution at 37 °C for 30 min before experimentation. Permeability was assessed electrophysiologically via measurement of transepithelial electrical resistance (TEER). The experimenter was blinded to the animal groups. In order to calculate the TEER, the potential difference (PD) and short circuit current (Isc) were recorded and TEER was calculated using Ohm's law as follows: I=PD/R.

### 3.7 Statistics and experimental rigor

The data is represented as mean ± SD. Analyses were performed on Prism 9 (www.graphpad.com). Paired t-tests, 1- or 2-way analysis of variance (ANOVA) with Bonferonni's post-hoc tests were used to analyze the results. Cohort sizes were determined based on previous studies with similar experimental designs. Two female animals did not participate adequately in the VMR test and 1 female died before day 28. All three were excluded from the final results (2 SH + SHAM and 1 SH + WAS).

## 4 Results

### 4.1 Brief EE exposure *following* WAS is sufficient to reverse persistent stress-induced visceral hypersensitivity in male and female rats

**Fig. 1:**
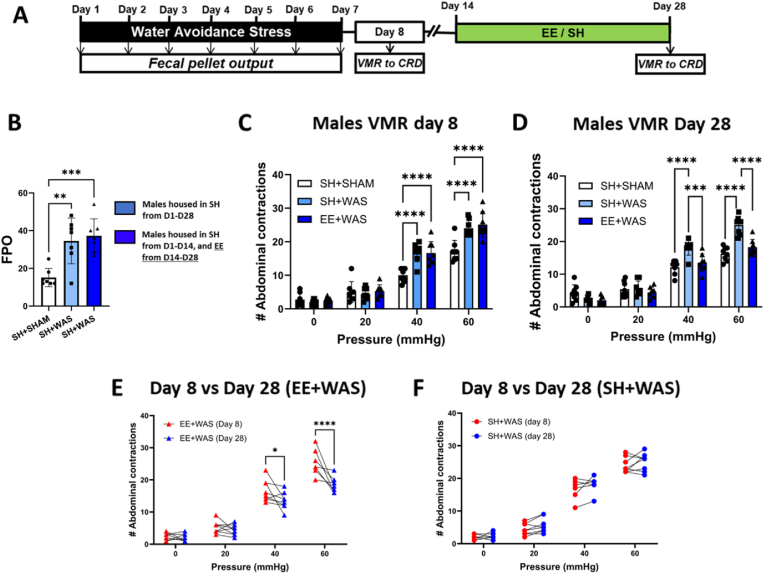
Exposure to EE reversed stress-induced visceral hypersensitivity in male rats: (A) Experimental Design. (B) WAS increased total FPO in male rats compared to SHAM (p < 0.001). (C) WAS caused increased abdominal contractions at 40 mmHg (p < 0.001) and 60 mmHg (p < 0.01) distension pressures on day 8. (D) Exposure to EE was able to reverse WAS-induced increase in abdominal contractions back to sham levels on day 28. (E) Stressed animals exposed to EE had significantly lower abdominal contractions on day 28 when compared to their day 8 values. (F) Stressed animals not exposed to EE were still as hypersensitivity on day 28 as day 8. B, one-way ANOVA, C–F, repeated measure two-way ANOVA. ***p < 0.001, ****p < 0.0001.

**Fig. 2:**
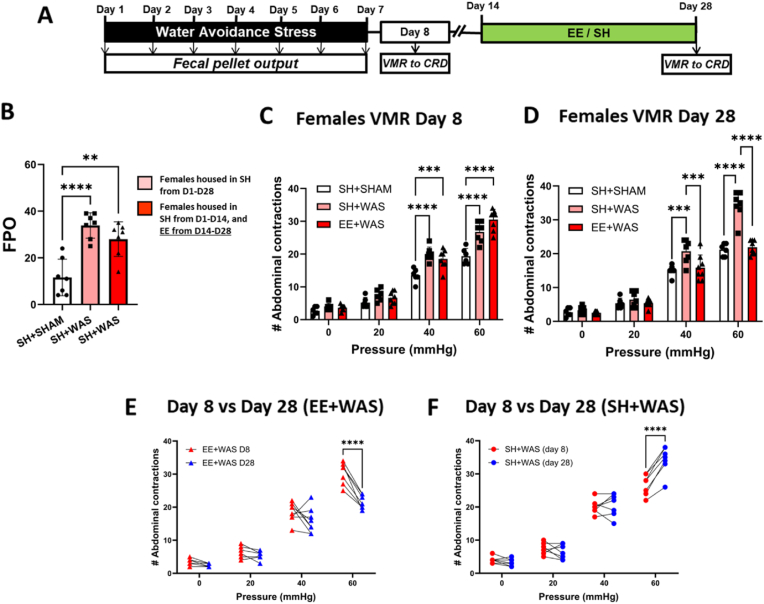
Exposure to EE reversed stress-induced visceral hypersensitivity in female rats: (A) Experimental design. (B) Female rats exposed to WAS had a significantly higher FPO compared to rats in the SHAM group. (C) WAS induced increased abdominal contractions at distension pressures of 40 mmHg (p < 0.001) and 60 mmHg (p < 0.0001). (D) EE was sufficient to reverse stress-induced visceral hypersensitivity in female rats. (E) Stressed females exposed to EE were significantly less hypersensitive on day 28 compared to day 8. (F) Stressed females not exposed to EE were even more hypersensitive on day 28 when compared to their day 8 values. B, one-way ANOVA, C–F, repeated measure two-way ANOVA. ***p < 0.001, ****p < 0.0001.

The experimental design for this series is shown in Fig. 1, Fig. 2A. Exposure to daily WAS caused increased total FPO compared to sham controls in both males (F_(2, 18)_ = 12.20; p = 0.0004; Fig. 1B) and females (F_(2, 18)_ = 19.10; p < 0.0001) (Fig. 2B). Visceral sensitivity was assessed 24 h after the final WAS procedure (day 8). Two-way repeated measure (RM)-ANOVA of visceral sensitivity measurements in rats revealed a main pressure effect (Males: F_(3, 63)_ = 531.6; p < 0.0001; Females: F_(3, 54)_ = 577.2; p < 0.0001), a main group effect (Males: F_(2, 21)_ = 8.694; p < 0.0001; Females F_(2, 18)_ = 22.65; p < 0.0001) and a group × pressure interaction effect (Males: F_(6, 63)_ = 13.32; p < 0.0001; Females: F_(6, 54)_ = 11.31; p < 0.00.0001). Bonferroni post hoc tests showed that WAS caused a significantly higher number of contractions when compared to sham controls at 40 mmHg (Males: p < 0.0001; Females: p = 0.004) and 60 mmHg (Males: p < 0.0001; Females: p = 0.001; Fig. 1, Fig. 2C). A cohort of stressed animals was then housed in EE for 2 weeks (days 14–28; Fig. 1, Fig. 2A) and visceral sensitivity assessed again 3 weeks after cessation of WAS (day 28). Two-way RM-ANOVA and Bonferroni post hoc tests revealed that stressed animals who stayed in standard housing (SH + WAS) were still hypersensitive when compared to sham controls at distension pressures of 40 mmHg (Males: p < 0.0001; Females: p = 0.0095) and 60 mmHg (Males: p < 0.0001; Females: p = 0.0001) (Fig. 1, Fig. 2D). Similar to the day 8 results, there were main effects for pressure (Male: F_(3, 63)_ = 541.5; p < 0.0001; Females: F_(3, 54)_ = 683.6; p < 0.0001), group (Male: F_(2, 21)_ = 10.79; p < 0.0006; Females: F_(2, 18)_ = 18.88; p < 0.0001), and the interaction (Male: F_(6, 63)_ = 17.83; p < 0.0001; Females: F_(6, 54)_ = 21.42; p < 0.0001). However, stressed animals which were exposed to EE (EE + WAS) showed significantly reduced the VMR scores compared to SH + WAS animals at 40 mmHg (Males: p < 0.0001; Females: p = 0.05) and 60 mmHg (Males: p < 0.0001; Females: p = 0.0002) (Fig. 1, Fig. 2D). EE + WAS VMR on day 28 was also significantly lower than their day 8 VMR scores pre-EE exposure (60 mmHg (Males: p < 0.0001; Females: p = 0.0002) Fig. 1, Fig. 2E). And were comparable to sham controls. SH + WAS animals were still hypersensitive at day 28 (Fig. 1, Fig. 2F).

### 4.2 Brief EE exposure following WAS reversed persistent stress induced somatic hypersensitivity in male and female rats

**Fig. 3:**
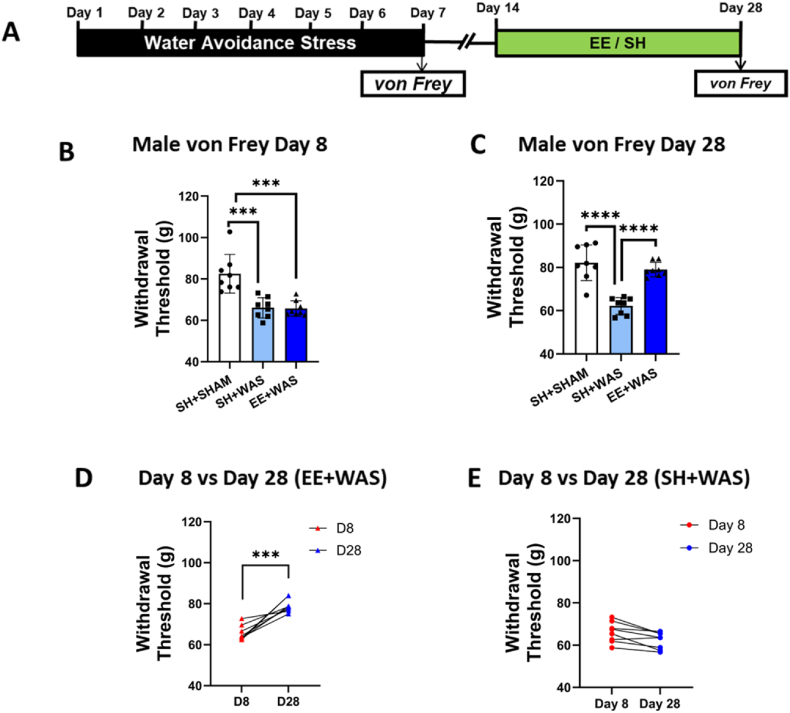
EE exposure reversed stress-induced somatic hypersensitivity in male rats: (A) Experimental design. (B) WAS causes a significant decrease in withdrawal threshold in male rats (p < 0.001). (C) Exposure to EE was sufficient to reverse stress-induced somatic hypersensitivity in male rats back to control levels. (D) EE significantly increased the withdrawal threshold of stressed male rats on day 28 compared to their day 8 values (p < 0.001). (E) Stress animals not exposed to EE were still hypersensitive on day 28 when compared to day 8. B, C, one-way ANOVA, D, E, paired Student's *t*-test. ***p < 0.001, ****p < 0.0001.

**Fig. 4:**
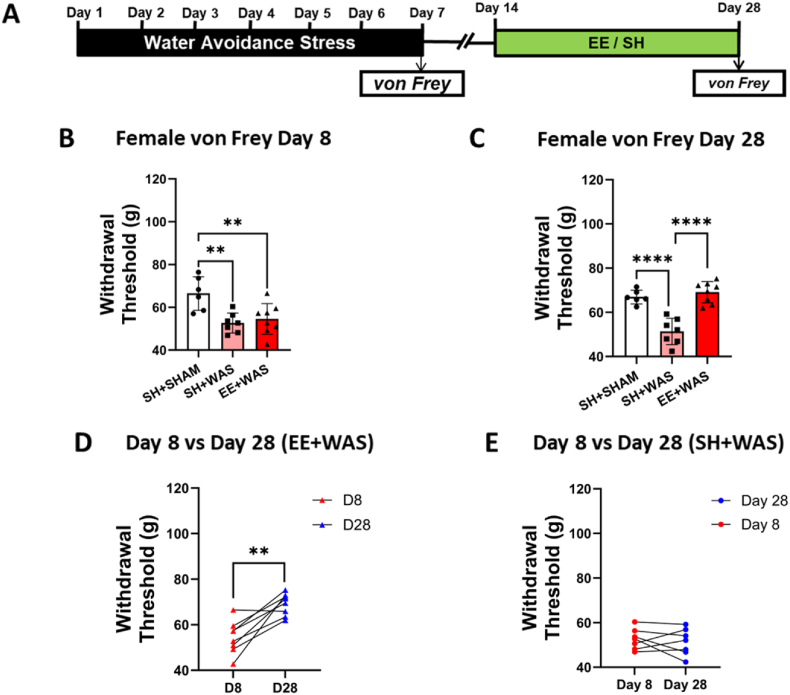
EE reversed stress-induced somatic sensitivity in female rats: (A) Experimental design. (B) WAS induced somatic hypersensitivity in female rats when compared to SHAM controls (P < 0.01). (C) EE reversed stress-induced somatic hypersensitivity restoring withdrawal thresholds back to control levels. (D) Exposure to EE significantly increased the withdrawal threshold of stressed females on day 28 compared to day 8. (E) Stressed animals not exposed to EE had similar withdrawal thresholds on both day 8 and day 28. B, C, one-way ANOVA, D, E, paired Student's *t*-test. **p < 0.01, ****p < 0.0001.

Due to the comorbidity of somatic and visceral pain disorders, we evaluated somatic sensitivity. Somatic sensitivity was assessed on day 6 after the WAS procedure (Fig. 3, Fig. 4A). One-way ANOVA with Bonferroni post hoc analysis revealed that stressed animals also had significantly lower withdrawal threshold compared to controls for both males (F_(2, 21)_ = 17.64; p = 0.0001) (Fig. 3B) and females (F_(2, 18)_ = 7.981; p = 0.0033) (Fig. 4B). After exposing the EE + WAS group to an enriched environment for 2 weeks, somatic sensitivity was once again assessed on day 28. 3 weeks after the end of the WAS procedure, we measured a persistent somatic hypersensitivity in stressed males (F_(2, 21)_ = 29.31; p < 0.0001) (Fig. 3C) and females (F_(2, 18)_ = 28.49; p < 0.0001) (Fig. 4C) not exposed to EE. Stressed animals exposed to EE from days 14–28 had withdrawal thresholds that were comparable to controls and significantly higher than SH + WAS animals on day 28 (one-way ANOVA with Bonferroni, p < 0.0001) (Fig. 3, Fig. 4C). Paired t-tests also showed that EE + WAS animals had significantly higher withdrawal thresholds compared to their day 8 values (Males: t_(7)_ = 6.235; p = 0.0004; Females: t_(7)_ = 4.633; p = 0.0024) (Fig. 3, Fig. 4D). SH + WAS animals still displayed somatic hypersensitivity on day 28 (Fig. 3, Fig. 4E).

### 4.3 EE exposure ameliorates stress induced colonic hyperpermeability in both male and female rats

**Fig. 5:**
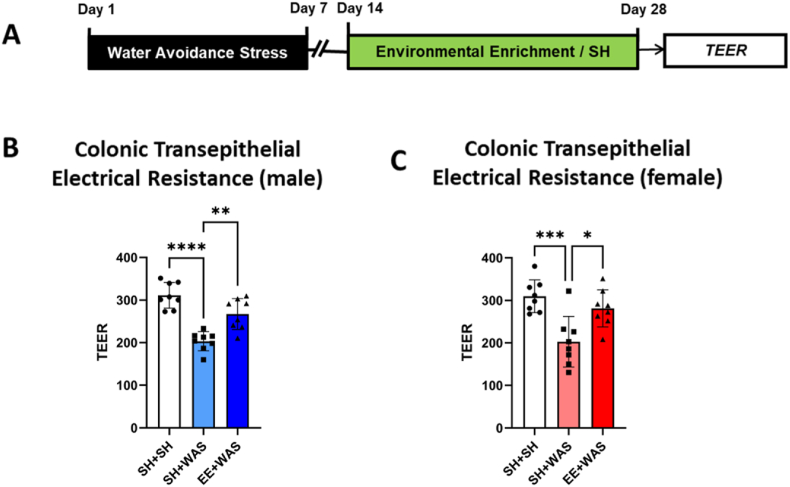
EE ameliorated stress-induced colonic hyperpermeability in both male and female rats: (A) Experimental design. (B) EE partially reversed stress-induced decrease in TEER in male animals back to SHAM control levels (p < 0.01). (C) EE was sufficient to reverse stress-induced decrease in TEER back to control levels (p < 0.05). B, C, one-way ANOVA. *p < 0.05, **p < 0.01, ***p < 0.001, ****p < 0.0001.

We measured transepithelial electrical resistance (TEER) as a measure of colonic permeability in a separate cohort of animals that did not undergo CRD on day 28 post WAS (Fig. 5A). One-way ANOVA analysis revealed significant differences between the groups in both male (F_(2, 38)_ = 34.14; p < 0.0001) and female (F_(2, 38)_ = 10.60; p = 0.0002) animals. Bonferroni post hoc tests showed that WAS caused a significant reduction in TEER (indicative of higher permeability) when compared to sham controls in both male (sham = 311.0 ± 35.9 Ωcm^2^ vs WAS = 206.5 ± 24.6 Ωcm^2^; p < 0.0001; Fig. 5B) and female (sham = 305.0 ± 41.3 Ωcm^2^ vs WAS = 217.1 ± 62.2 Ωcm^2^; p < 0.0002; Fig. 5C) animals which was still present on day 28. In contrast, exposure to EE was able to partially reverse stress-induced decrease in colonic TEER in both male (EE + WAS = 272.0 ± 36.4 Ωcm^2^ p < 0.0001) (Fig. 5B) and female (281.6 ± 48.1 Ωcm^2^; p < 0.0066) (Fig. 5C) animals.

### 4.4 EE exposure reverses stress-induced changes in GR expression at the CeA and hippocampus of male and female rats previously exposed to WAS

**Fig. 6:**
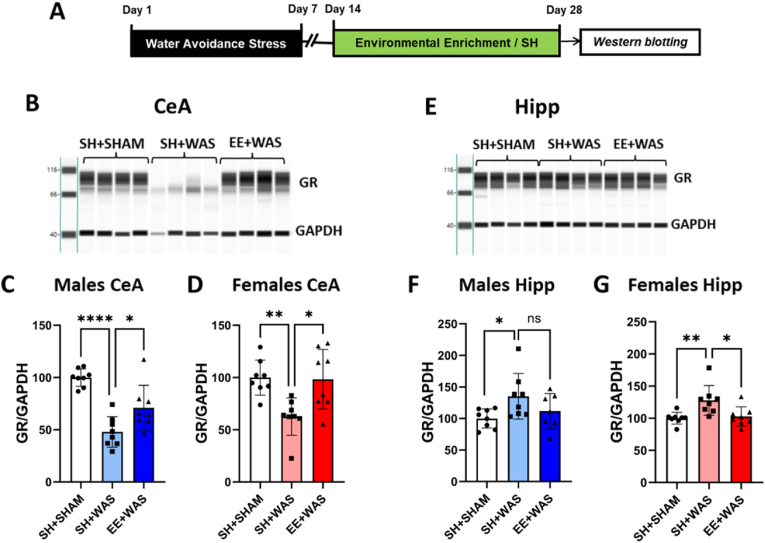
EE reversed stress-induced changes in GR expression in both the CeA and hippocampus: (A) Experimental design. (B) Digital representation of GR and GAPDH expression in the CeA of the different cohorts. EE exposure attenuated stress-induced decrease in GR in the CeA for both (C) males (p < 0.05) and (D) females (p < 0.01). (E) Digital representation of GR and GAPDH expression in the hippocampus of the different cohorts. EE exposure reversed stress-induced increase in GR expression in the hippocampus of both (F) males (p = 0.055) and (G) females (p < 0.05). C, D, F, G, one-way ANOVA. *p < 0.05, **p < 0.01, ****p < 0.0001.

We also measured expression levels of GR in the brain regions we had previously seen to be involved in stress induced visceral hypersensitivity (Fig. 6A and B). In the CeA, 1-way ANOVA revealed significant differences between the groups in males (F_(2, 21)_ = 21.9; p < 0.0001) and females (F_(2, 21)_ = 7.529; p = 0.0034). Bonferroni post-hoc tests showed that WAS caused significant long-term reduction in GR expression compared to sham controls in both males (sham = 100 ± 8.3 vs WAS = 47.9 ± 14.6; p < 0.0001) (Fig. 6C) and females (sham = 100 ± 16.8 vs WAS = 62.7 ± 18.0; p = 0.0048) (Fig. 6D). Exposure to EE was sufficient to attenuate WAS-induced reduction in GR in the CeA for both males (EE + WAS = 71.1 ± 21.5; p = 0.015) (Fig. 4C) and females (EE + WAS = 98.4 ± 28.6; p = 0.0067) (Fig. 4D). We also measured GR levels in the hippocampus, where we saw the opposite trend. One-way ANOVA revealed significant differences in hippocampal GR expression between the groups for female animals (F_(2, 21)_ = 6.749; p = 0.0055) (Fig. 6E). Using Bonferroni post-hoc tests revealed that WAS caused a significant increase in GR expression when compared to controls (sham = 100 ± 9.1 vs WAS = 127.9 ± 2.15; p = 0.006), but exposing a cohort of stressed animals to EE normalized GR expression back to control levels in the hippocampus of female rats (EE + WAS = 102.7 ± 14.9; p = 0.013) (Fig. 6G). In male animals, one-way ANOVA showed modest differences between the groups (F_(2, 20)_ = 3.319; p = 0.056) (Fig. 6F). Bonferroni's post-hoc tests showed an association between stress and increased GR expression in the hippocampus (sham = 100 ± 15.2 vs WAS = 135.2 ± 36.1; p = 0.039) and EE showed a trend of reducing GR levels back to normal however, this difference was not significant (EE + WAS = 111.8 ± 27.9; p = 0.11) (Fig. 6F).

## 5 Discussion

Our data indicates that in a well characterized rodent model, repeated exposure to a daily stressor induced chronic visceral and somatic hypersensitivity. Repeated water avoidance stress also induced multiple molecular changes that ultimately led to persistent visceral and somatic hypersensitivity in both males and females. Furthermore, exposure to an enriched environment was able to counter these effects of chronic stress. We showed that exposing animals to EE, a rodent analogue of behavioral therapies, for 2 weeks is sufficient to reverse the effects of pre-existing chronic stress. EE reverses stress-induced changes in the expression of the stress receptor (GR) in both the CeA and hippocampus as well as stress-induced changes in the colonic permeability to ameliorate stress-induced visceral and somatic hypersensitivity. Animals in the EE + WAS group showed visceral sensitivity that was comparable to controls and significantly lower than their previous levels before EE exposure or in WAS treated animals that were never moved to an enriched environment. These results were similar in both males and females indicating that EE is effective in both males and females.

Behavioral therapies including CBT and other stress management techniques have emerged as effective treatments for IBS symptoms including visceral pain (Kinsinger, 2017; Lackner et al., 2007, 2008) however, the molecular mechanisms are still unclear. In order to reveal the molecular changes behind the effects of behavioral therapies on IBS symptoms, we investigated the effects of rodent behavioral interventions on different components that are critical to the development of visceral and somatic hypersensitivity: GR expression in the CeA (and hippocampus) and colonic permeability.

Multiple clinical and preclinical studies have highlighted the importance of GR expression in the CeA in the pathophysiology of stress-induced visceral hypersensitivity. IBS patients show increased amygdala reactivity during colonic balloon distension (Wilder-Smith et al., 2004). The current study demonstrates that EE exposure was effective in reversing existing chronic stress-induced decreased GR expression without needing pre-exposure to EE before the stressor. The hippocampus also plays an active role in stress and anxiety management. A chronic increase in GR activation in the hippocampus is associated with a hyperactive HPA axis and visceral hypersensitivity (Zhu et al., 2014; Zhang et al., 2016). Here we add to the evidence that stress can increase GR expression in the hippocampus and exposure to EE is sufficient to reverse these stress-induced changes in rodents. Together, the stress-induced changes in GR expression at the CeA and hippocampus precipitate a hyperactive stress response axis and increased nociception (Jankord et al., 2008). Our data suggests that behavioral therapies can directly affect the molecular mechanisms of stress-induced visceral hypersensitivity to combat the symptoms.

Colonic integrity and permeability to luminal content can also influence nociception and visceral sensitivity (Zhou et al., 2009), which led us to investigate whether stress and EE also had an effect on colonic integrity. Chronic stress has also been shown to downregulate tight junction proteins and increase colonic permeability (Zheng et al., 2013). We showed that animals in the WAS group had significantly higher colonic permeability compared to the control and EE groups despite the stressor (WAS) and intervention (EE) both being psychological and not directly targeting the colon. The exact mechanism by which these psychological interventions are affecting colonic integrity is unknown. There is a possibility that EE has a direct effect on colon tissue by affecting tight junction expression and permeability or through an indirect effect. We previously observed that stress can sensitize brain-gut communication at the level of the spinal cord (Orock et al., 2021b). Sensitized nociception at the level of the dorsal horn where somatic and visceral pathways converge could explain why somatic hypersensitivity is sometimes comorbid with visceral hypersensitivity and IBS patients usually also present with somatic disorders like fibromyalgia. Hence, it is possible that the activated spinal pathway also sensitizes the enteric nervous system and activates inflammatory markers that disrupt colonic integrity. There are other factors including active colitis (Gracie et al., 2015; Ozdil et al., 2011) and post infectious colonic sensitization (Thabane et al., 2009) that also induce visceral hypersensitivity. The fact that exposure to EE was able to reverse colonic hyperpermeability suggests that behavioral therapies can be effective therapies for colitis-induced IBS disorders (either on their own, or in combination with pharmaceuticals). Further studies into the effects of stress on specific tight and gap junction proteins, inflammatory/immune mediators and enteric neuron sensitization, are required to reveal the specific roles they play in the induction and maintenance of visceral hypersensitivity.

We noticed from our data that while the male WAS animals maintained similar levels of hypersensitivity on days 8 and 28, females seemed to be more hypersensitive on day 28 compared to their day 8 values and compared to males. This potentiation of hypersensitivity over time may explain why the disorder shows a female-predominant phenotype as female symptoms may get worse over time leading to an increased number of diagnoses. Future studies are needed where the females and estrus cycles are taken into consideration to confirm this sexual predominant effect. Exposure to EE was able to bring the colonic sensitivity values of animals in the WAS group back to sham control levels for both males and females despite the difference in abdominal contraction scores between the groups on day 28.

It should be noted that while we focused on the stress “brain-gut” pathway for this study, EE and behavioral therapies also affect multiple regions of the brain making it difficult to study all the associated molecular changes. We used a more focused approach and only studied regions we knew played a role in visceral sensitivity; however, it is possible that EE also acts through additional unexplored central pathways.

*In conclusion,* our data shows that placing animals with stress-induced visceral hypersensitivity in an EE for 2 weeks is sufficient to reverse stress-induced changes in GR expression at the CeA and hippocampus. EE also reversed stress-induced colonic hyperpermeability and ultimately ameliorated stress-induced visceral and somatic hypersensitivity in both males and females. Translationally, this study shows that behavioral therapies can work by counteracting the molecular and physiological effects of stress that lead to visceral and somatic hypersensitivity meaning it is not a placebo effect. Additionally, EE was effective without the need to “pre-expose” the animals to EE before the stressor, suggesting that behavioral therapies may have positive effects without the need to “pre-expose” the patient to the therapy before the trigger/stressor. This significantly improves the therapeutic avenues of behavioral therapies. We also showed that EE is equally effective in both males and females suggesting behavioral therapies should work in both sexes despite the sexually dimorphic nature of the disorder.

## CRediT authorship contribution statement

**A. Orock:** Methodology, Investigation, Writing. **A.C. Johnson:** Supervisor, Formal analysis, Writing – review & editing. **E. Mohammadi:** Methodology, Validation. **B. Greenwood-Van Meerveld:** Conceptualization, Project administration, Funding acquisition, Supervision.

## Declaration of competing interest

The authors declare that they have no known competing financial interests or personal relationships that could have appeared to influence the work reported in this paper.
